# Severe Hyperacusis, Photophobia, and Skin Hypersensitivity

**DOI:** 10.1155/2016/2570107

**Published:** 2016-02-15

**Authors:** Alessandra Barbara Fioretti, Theodoros Varakliotis, Otello Poli, Manuela Cantagallo, Alberto Eibenstein

**Affiliations:** ^1^Tinnitus Center, European Hospital, Rome, Italy; ^2^Department of Applied Clinical Sciences and Biotechnology, L'Aquila University, Via Vetoio (Coppito 2), 67100 Coppito, Italy

## Abstract

We report a case of a patient with severe hyperacusis, photophobia, and skin hypersensitivity. The patient was initially treated with sound therapy and medical therapy for 4 months and successfully with a selective serotonin reuptake inhibitor (SSRI) and cognitive behavioral therapy which improved her mood and the tolerance for sounds and light.

## 1. Introduction

Hyperacusis is defined as “unusual tolerance to ordinary environmental sounds” [1] or as “consistently exaggerated or inappropriate responses to sounds that are neither threatening nor uncomfortably loud to typical person” [2]. Hyperacusis should be distinguished from photophobia (fear of sound) and misophonia (dislike towards specific sounds) [3], which are associated with a strong negative emotional response. It is suggested that the term of hyperacusis, is used for various types of hearing hypersensitivity and not for a specific range of sounds. Recruitment is frequently confused with hyperacusis, it is an increased loudness perception [4] caused by a dysfunction of the outer hair cells (OHC) with cochlear hearing impairment. Recruitment is defined as an abnormal increase in the sensitivity to increasing loudness of sound in the affected ear. This phenomenon of recruitment can be used to distinguish between cochlear and retrocochlear impairment. If the hearing is normal, hypersensitivity is always due to hyperacusis (plus or minus misophonia) and never due to recruitment.

It is widely noted that patients suffering from tinnitus also present hyperacusis in 40% of cases [5–7]. Instead, in patients that have been diagnosed with hyperacusis, tinnitus has been reported in 86% [8].

Hyperacusis may be associated with ear pathologies like Ménière's disease, perilymphatic fistula, sudden sensorineural hearing loss, acoustic trauma, otosclerosis, Bell's facial palsy, and Ramsay Hunt syndrome. In addiction, there is a correlation with CNS disorders like migraine, depression, posttraumatic stress disorders, multiple sclerosis, benign intracranial hypertension, Tay-Sachs syndrome, Williams syndrome, and Lyme's disease.

The differential diagnosis between recruitment and hyperacusis is based on audiologic routine tests like tonal and speech audiometry, tympanometry, and acoustic middle ear reflex examination. Auditory Brainstem Responses (ABR) are useful for a differential diagnosis for retrocochlear diseases, vestibular schwannoma, and neurovascular conflict, together with MRI and angioMRI. There are also specific tests for hyperacusis such as the determination of the loudness discomfort level (LDL), which is generally considered pathological at a threshold below 90 dB HL [9]. Questionnaires for decreased sound tolerance are useful in the clinical diagnosis and have been developed in these years [10]. With these questionnaires it is possible to evaluate cognitive reactions to hyperacusis, behavioral changes, and emotional responses to external sound [11]. The questionnaire published from Khalfa evaluates also attention, social interaction, and emotions [12].

## 2. Case Report

A female patient, 72 years old, came to our attention complaining of a chronic hyperacusis that occurred almost one year ago. During the evaluation, the patient described her hyperacusis as an increased sensibility to environmental sounds. This sensation was accompanied to an intense discomfort after exposure to sounds and to loud voices too. A particular association with a widespread skin hypersensitivity and a visual intolerance to intense light was revealed. Hyperacusis was evaluated with uncomfortable loudness levels (ULL) measured at 0.25 kHz, 0.5 kHz, 1 kHz, 2 kHz, 3 kHz, 4 kHz, and 6 kHz.

**Figure 1:**
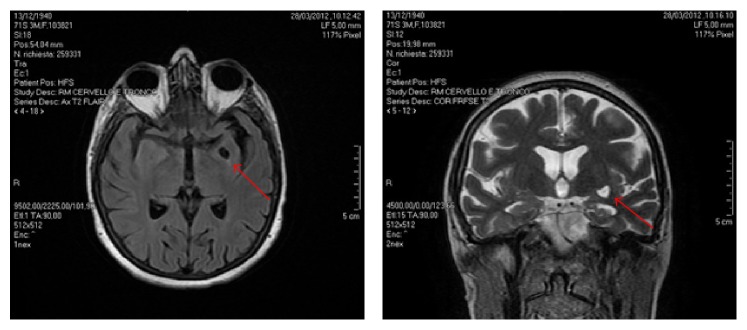
An expanded round cavity with liquid content and thin halo of gliosis was evidenced with MRI (indicated with arrows).

She referred hypertension and hypothyroidism in treatment. In March 2012, an MRI was performed which indicated an expansion of the supratentorial ventricular system prevailing in the frontal region. The left temporal region featured an expanded round cavity with liquid content and thin halo of gliosis, with a simultaneous atrophy of the temporal, limbic, and hippocampal structures more prominent on the left side (Figure 1).

In May 2012, she was examined at the European Hospital Tinnitus Center for a hearing disorder characterized by hyperacusis and photophobia, photophobia, and skin hypersensitivity. These disorders were associated with agoraphobia, mood deflection, hypomimia, skin hyperaesthesia, and absence of ofthalmological pathologies. In the tests of manual dexterity there were not evident motor disorders. Deambulation was cautious, attitude with the trunk bent forward.

**Figure 2:**
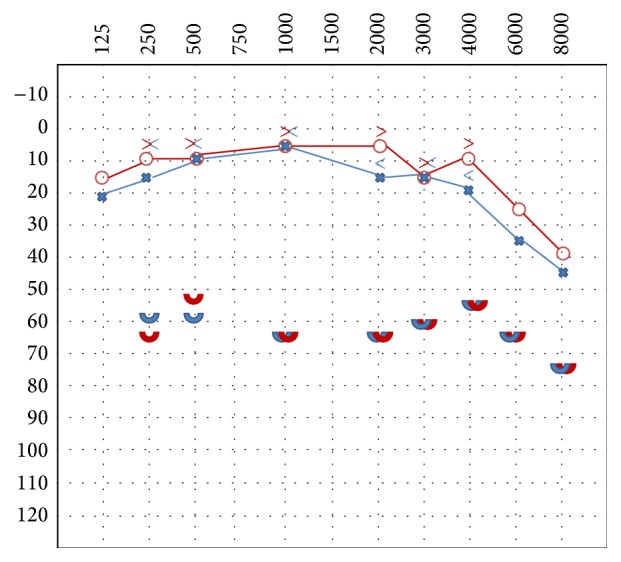
Audiological test with ULL showing severe hyperacusis before the treatment.

The onset was rapid, noted in June 2011 as a result of cataract surgery in local anesthesia. The following questionnaires were carried out: Pittsburgh Sleep Quality Index (PSQI): 7 (mild sleep disorder) and hyperacusis questionnaire of Khalfa: 36 (positive for hyperacusis). The tonal audiometry showed a mild sensorineural hearing loss in the high frequencies bilaterally and more marked on the left. The ULL showed abnormal levels of tolerance (severe hyperacusis) at levels of 55–65 dB HL symmetrical in both ears (Figure 2). The impedance test indicated a normal compliance with Jerger tympanogram type A bilaterally. The stapedial reflexes were not performed due to decreased tolerance to sound. The distortion products of otoacoustic emissions (DPOAE) resulted normal bilaterally for 2, 3, and 4 kHz frequencies. A very fast response time should be noted. Our conclusion was that the patient suffered from hyperacusis and misophonia (Jastreboff category 4).

A standard EEG emphasized irritative abnormalities on the frontotemporal lobes, more evident during the hyperpnea, in the context of a dysrhythmic track, unstable and irregular.

**Figure 3:**
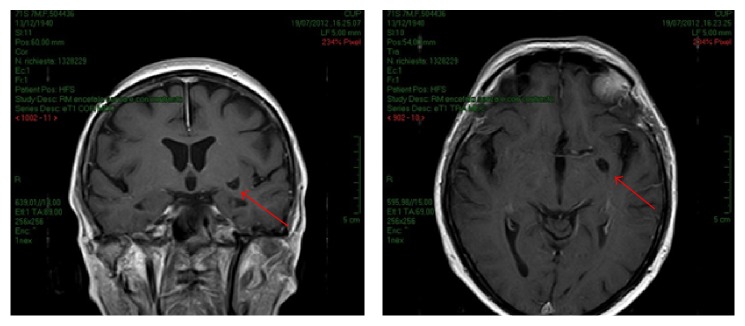
A large CSF perivascular space of the anterior perforated substance, in the basal portion of the left lentiform nucleus evidenced by a second MRI (indicated with arrows).

In July 2012, she repeated the MRI which indicated large CSF perivascular space of the anterior perforated substance, in the basal portion of the left lentiform nucleus and occasional dilated CSF perivascular space without pathological significance (Figure 3).

The psychological assessment revealed deep psychological distress characterized by anxiety and expressed through various physical symptoms. The patient, deeply depressed and sad, has a representation of the world as hostile and threatening, from which she cannot defend herself. The behavior was characterized by pessimism, doubtfulness, passivity, and fear of expressing aggressive or sexual feelings. The subject tends to subordinate her needs to those of others, delegating to them the responsibility for the key areas of her life; she shows submission and lacks in self-confidence.

She is very self-critical and feels strong guilt emotions. The results of psychological tests are summarized in Table 1 [13–16]. The Millon Clinical Multiaxial Inventory-III was invalid because 17 items were without answer.

The patient was treated with Pregabalin, Levetiracetam, and bilateral sound generators with initial benefit.

Bilateral sound generators were used initially set at just audible level, progressively increasing the level of sound and subsequently changing volume as necessary. The objective was the reduction in anxiety and fear. More specifically for the patient's hyperacusis disorder, a program was proposed for the desensitization of the auditory system (for the reduced tolerance to sounds) and for misophonia another was adviced for active listening to pleasant sounds.

**Figure 4:**
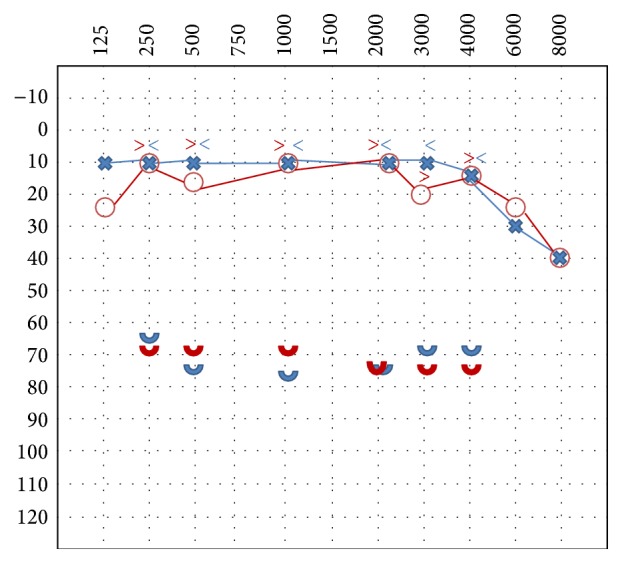
Audiological test with ULL showing moderate hyperacusis after the treatment.

The psychologist prescribed a psychiatric examination, cognitive behavioral psychotherapy, or psychological counseling for at least 4–6 months but the patient did not follow the recommended psychological therapy. After 4 months, the patient revealed reduced symptoms related to hyperacusis and photophobia but in July 2012 she suspended the pharmacological therapy because she was treated for an invasive ductal cancer (T2 N1 M0) with mastectomy and chemotherapy. In the follow-up after 8 months, at the end of chemotherapy the patient reported a worsening of hyperacusis and photophobia. She started a therapy with a selective serotonin reuptake inhibitor (SSRI) and cognitive behavioral therapy for 4 months which improved her mood and the tolerance for sounds and light. In October 2014, the patient improved hyperacusis and photophobia while skin hypersensitivity disappeared. The score of hyperacusis questionnaire of Khalfa was 14 (negative for hyperacusis). The tonal audiometry showed a mild sensorineural hearing loss in the high frequencies bilaterally. The ULL showed abnormal levels of tolerance (moderate hyperacusis) at levels of 65–75 dB HL symmetrical in both ears at all frequencies (Figure 4).

## 3. Discussion

We described for the first time the association between hyperacusis, skin hypersensitivity, and photophobia in a patient.

The acoustic cortex is localized on upper temporal gyrus and the somatosensory cortex is situated on postrolandic parietal gyrus. The lesion in left temporal lobe of the patient was causative of abnormal bioelectric discharges recorded with EEG.

Probably the association pathway between acoustic cortex, where the lesion is localized, and somatosensory cortex could explain the diffusion of irritative status such as a deficit of inhibitory action of CNS.

The locus ceruleus, situated in the brainstem underfloor of IV ventricle, is the main nervous centre of synthesis and release of noradrenaline. It is formed from groups of noradrenergic neuronal cells.

Noradrenaline has an exciting effect in brain and modulates the pain awareness.

Deficit of inhibitory control of CNS on locus ceruleus should have played an important role in all disorders.

Furthermore we have to consider that sensory stimulation like hearing, vision, smell, taste, and touch increases excitation of locus ceruleus neuronal cells.

In consideration of the age and the depression of the patient, we considered the role of serotonin in the modulation of auditory signals and we prescribed SSRI. As reported by Cruz et al., SSRI may improve the results of auditory processing in elderly patients (Cruz OL, Kasse CA, Sanchez M, Barbosa F, and Barros FA; serotonin reuptake inhibitors in auditory processing disorders in elderly patients: preliminary results. Laryngoscope 2004; 114:1656-9).

There is a valid hypothesis about nonsyndromic hyperacusis related to the patient. Dysfunction of the medial efferent system might lead to reduced damping of the cochlea. This can happen in case of peripheral lesions and the neuroplasticity of the ascending way. Stress causes the release of endogenous opiates or dynorphins in the inner hair cells (IHC). This could increase the cochlear neurotransmitter glutamate, which might lead to enhanced auditory nerve activity. An auditory signal activates an abnormal process of amplification in subconscious auditory pathways, causing secondary activation of the limbic and autonomic nervous systems.

Hwang et al. studied 3 patients with hyperacusis by fMRI and they found that the frontal lobes and parahippocampus might be associated with phobic hypersensitivity to unpleasant sounds in patients with idiopathic hyperacusis [17]. The brain activation studied with fMRI could clarify the areas involved in our patient.

Hyperacusis is common among severely affected patients with complex regional pain syndrome related dystonia. Hyperacusis in these patients may reflect the spreading of central sensitization to auditory system [18]. Finally, as reported by Hasson et al., women with high levels of emotional exhaustion become more sensitive to sound after an acute stress task and had reduced thresholds to loudness. Patients with normal ULL but seeking help for hyperacusis should be assessed for emotional exhaustion (plasma cortisol concentration, estradiol) [19].

## 4. Conclusion

There is a close relationship between hyperacusis, hypersensitivity, and pain.

In this clinical case, the treatment has had an effective equilibrating action. As for chronic pain, a multidisciplinary approach is necessary for effective results. The appropriate hyperacusis sound therapy and counseling protocol must be applied for the desensitization of the auditory system. In case of a multisensory disorder (auditory, visual, and somatosensory) a pharmacological and psychological support is indicated.

## Figures and Tables

**Table 1:** Scores obtained by the patient on psychological tests.

Symptom checklist 90-R (SCL-90-R)		
Somatization	1.9	
Obsessive-compulsive	1.4	
Interpersonal sensitivity	0.2	
Depression	1.7	
Anxiety	1.8	
Anger-hostility	2	
Phobic anxiety	0.1	
Paranoid ideation	0	
Psychoticism	1.5	
Sleep disorders	4	
Eysenck Personality Questionnaire		
Neurocriticism	22/27	High level
Psychoticism	8/30	Low level
Extroversion	15/25	Medium level
Lies	17/23	High level
Beck Depression Inventory (BDI)		
Somatic-affective	10/27	Down level
Cognitive	10/36	High level
Total	20/63	High level
State Trait Anxiety Inventory, X1 and X2 (STAI X1 and X2)		
State anxiety	71	Top level
Trait anxiety	55	Medium to high
